# Distribution of SNSs in Mimivirus Genomes and the Classification of Mimiviruses Isolated from Japan

**DOI:** 10.1264/jsme2.ME19077

**Published:** 2019-12-27

**Authors:** Motohiro Akashi, Masaharu Takemura

**Affiliations:** 1 Laboratory of Biology, Department of Liberal Arts, Faculty of Science, Tokyo University of Science Kagurazaka 1–3, Shinjuku, Tokyo 162–8601 Japan

**Keywords:** Mimiviridae, Giant virus, SNSs, fractal, Power law

## Abstract

Mimiviruses have been detected in various habitats. Analyses of single nucleotide substitutions (SNSs) have revealed that SNSs are mainly localized on both ends of the mimivirus genome, and mimivirus lineage A has been split into three genotype groups; therefore, mimiviruses may be classified into lineages and genotype groups based on SNSs. We isolated 9 mimiviruses from Japan and analyzed SNSs. These isolates were classified as lineage A genotype group type 2, suggesting that the local diversity of members of the family *Mimiviridae* isolated from *Acanthamoeba* spp. is lower than that of giant viruses from other families isolated in Japan.

Nucleo-cytoplasmic large DNA viruses (NCLDVs) comprise several groups called “giant viruses”, which are highly diverse (1, 10, 19). Among them, members of the family Mimiviridae have been discovered in various aquatic environments worldwide, such as seawater, rivers, lakes, and soils (11, 28). In addition to *Acanthamoeba polyphaga mimivirus* (APMV), which was the first mimivirus to be isolated (16, 21), more than 100 related mimiviruses have since been isolated globally (11, 28). Molecular phylogenetic analyses have classified these mimiviruses into two groups: group I (including APMV, megavirus, and moumouvirus) and group II (including *Cafeteria roenbergensis virus*). Group I viruses have been further sub-classified into three lineages: A (including APMV, mamavirus, and sambavirus), B (including *Megavirus chilensis*), and C (including moumoivirus) (11, 20, 28). Although the mimiviruses of lineage A exhibit the highest intra-lineage diversity, viruses belonging to lineages B and C have also been isolated in large numbers from seawater and land from various parts of the world, including Europe, Brazil, India, Antarctica, and Japan (2, 5, 8, 11, 16, 20, 21, 25, 28). The local diversification of this group in each country or local region remains to be elucidated. To address this gap in knowledge, we analyzed the SNSs distributed across the whole genomes of members from group I of the family Mimiviridae. The coding sequences of giant viruses were previously shown to be under negative selection, which suggests that giant virus genomes are highly stable, and SNSs on these genomes may accurately reflect their intra-familial relationships (12).

We obtained the genomic sequences of viruses belonging to group I of the family Mimiviridae from the NCBI genome database. The accession numbers of 37 sequences are listed in Data S1. SNSs were analyzed using MUMmer ver. 3.23 (15), with the APMV genome (NC_014649.1) as a query sequence. Comparisons of mimivirus genomes against the APMV genome were initially performed using the “nucmer” program on MUMmer. SNSs were detected using “show-snps” with the “−c” option (the −c option eliminates SNPs/SNSs from alignments with ambiguous mapping). In the present study, we detected approximately 100 k of segregating positions on the APMV genome (Table S1), and 915 (96.9%) CDSs on the APMV genome harboring SNSs (Table S1).

SNSs on each CDS of the APMV genome were counted and the SNS-CDS ratio (defined as SNS species/CDS length) was calculated, as described in the legend of Fig. S1. The mean SNS-CDS ratio was 0.0872, and the intergenic SNS ratio was 0.0838, indicating no significant difference in SNS density between CDS and non-CDS regions. In contrast, the SNS-CDS ratio correlated with the physical position of the CDS (Fig. S1). We plotted SNS-CDS ratios against the CDS-ID that we had labeled based on CDS locations from the 5′ end to the 3′ end of the APMV genome record (NC_014649.1). The SNS-CDS ratios in the 5′ and 3′ ends of the APMV genome exhibited larger values than those in the central positions of the APMV genome (Fig. S1A and B). Intergenic regions were previously considered to exhibit a higher substitution rate than genic regions because they are anticipated to be subjected to lower selective pressure than genic regions. However, we found that the frequency of substitutions between these regions of the genomes of viruses from Mimiviridae was similar. This result indicates that intergenic regions in the genomes of members from Mimiviridae have important functions. One of the possible functions of intergenic regions is the regulation of transcription. In eukaryotes, histones regulate transcription through histone methylation and enhancer sequences. Histone and histone-related eukaryotic genes are conserved in some viruses, including NCLDVs. For example, Lausannevirus and Marseillevirus encode eukaryotic/archaeal histones (26). All five types of histones (H1, H2A, H2B, H3, and H4) were recently shown to be conserved in medusavirus (30). Furthermore, mimiviruses do not conserve histones themselves, but harbor putative histone demethylases (YP_003987130.1 in the NCBI Protein database). Therefore, mimiviruses appear to regulate the expression of their genes through histone-methylation/demethylation. The regulation of mimivirus gene expression may be more strictly controlled by intergenic regions and histones than in eukaryotes based on the low substitution rates in their intergenic regions. Furthermore, we found that the histogram of SNS-CDS ratios showed a skewed distribution; a high ratio was localized at both extremities of the APMV genome (Fig. S1). These results suggest that SNSs are not randomly distributed throughout the whole genome; however, their locations are dependent on the genomic structure. This may be attributed to nucleotide substitutions during DNA replication. We speculate that two processes contributed to this result. The first hypothesis is that DNA replication forks of mimiviruses start in the middle and proceed towards both ends. The hypothetical DNA replication origin of a mimivirus, estimated based on the AT skew of its genome, was located at 382.7 kb on its 1.18-Mb genome (3). As a result, the middle region of the genome exhibited lower substitution rates than the ends because the activity of the enzyme decreased with an increase in its denaturation. The second hypothesis is that both ends of the genome may frequently undergo recombinational repair. Although mimivirus genomes were previously reported to be linear, they have been speculated to form circular DNA (9), or both of their ends may conjugate for the replication of their terminal regions (29). Based on these hypotheses, the 5′ and 3′ end regions of mimivirus genomes repeatedly conjugate and get repaired during DNA replication. This frequent repair may lead to the higher substitution rates in these 5′ and 3′ end regions than in the middle region. The distribution of distances between SNSs in the human and yeast genomes has been reported to be approximated by the power law. The shape of regions including SNSs has been shown to exhibit a fractal structure (14). We tested whether the genomes of members from the family Mimiviridae exhibited the same characteristics using previously described methods (14). The positions of the 101,786 SNSs detected were used to confirm the distribution of distances between them. The frequencies of the distances at four different resolutions (1, 10, 100, and 1,000 bp) were counted and plotted on log-log scale graphs (Fig. S2). Frequency-distribution graphs included a scaling region that obeyed a power law (Fig. S2).

We also checked the fractal nature of the SNS distribution using the box-counting method (14). In this analysis, 46 resolutions (frames) were used (1≤r≤9, 10≤r≤90, 100≤r≤900, 1,000≤r≤9,000, 10,000≤r≤90,000, and r=100,000), and the total number N(r) of a given frame range (r) containing SNS(s) was counted and plotted on a log-log graph (Fig. S3). The resultant graph showed two scaling regions, which may be approximated by a power law. These were found in regions with resolutions lower and higher than 10 bp, and the slopes of each of the approximate lines were equal to the fractal dimensions 0.23 and 0.97, respectively (Fig. S3B and C). If segregation sites are selected randomly, the frequencies of distances between the SNSs show a uniform distribution; otherwise, they do not (Fig. S2). Therefore, this result indicates that SNS segregation sites are fractally localized and form SNS “hotspots” on the mimivirus genome, similar to the human/yeast genome. The mechanisms underlying the formation of SNP/SNS hotspots remain unclear; however, studies at the molecular level may resolve this issue.

*Mimivirus shirakomae* and *M. kasaii* were isolated from a freshwater pond and river in Japan, respectively (25). Only 19 SNSs were detected in the *M. kasaii* (*M. shirakomae*: 18 SNSs) genome relative to the APMV genome. Among these 19 loci, 9 were located in CDSs, with 8 representing non-synonymous substitutions (Data S2). Among the 19 SNSs and 27 spontaneous mutations previously reported in APMV (17), no overlapping positions were found. Since most of these CDSs harboring SNSs are predicted genes with unknown functions, we estimated their functions by homology modeling using the I-TASSER server (27). The results obtained suggest that these CDSs encode proteins related to cellular functions; *e.g*. ribonuclease, L-amino acid oxidase, and nucleoporin proteins (Data S3-1 and S3-2). We then performed a heat map clustering analysis to elucidate the detailed relationship between the two mimiviruses isolated in Japan, *M. shirakomae* and *M. kasaii*, and other lineage A members of the family Mimiviridae isolated from various aquatic environments worldwide (Table S2). The 19 positions of SNSs harbored within *M. shirakomae* and *M. kasaii* were extracted from the SNS matrix. The nucleotides A, T, G, and C were substituted by the numerical values 0, 1, 2, and 3, respectively, and are shown using colors ranging from black to red (Fig. 1). A heat map was drawn using the heatmap.2 function of the gplots package with R software (https://www.r-project.org). The heatmap clustering analysis of these 19 loci in members from lineage A of the family Mimiviridae formed three genotype groups, named here as “types 1, 2, and 3” (Fig. 1). In a previous study, lineage A was classified into two groups based on major capsid protein (MCP) genes (6). The four species (Kroon, Sangsue, Hal.V, and terra2) harboring distinct MCP genes were clustered into genotype group type 2 (Fig. 1). Moreover, APMV and two Japanese mimiviruses, which are genotypes harboring identical MCP genes, were classified separately into genotype group types 1 and 2, respectively (Fig. 1). To confirm the independency of genotype group types 2 and 3 from genotype group type 1, we computed pairwise distances based on pairwise sequence identities among species from the SNS matrix using the “dist.alignment” function in the “seqinr” package of the R program (7). The pairwise distances of all species, relative to the members of genotype group type 1 (APMV and APMV2), were grouped based on the three genotype groups; the significance of differences among these groups was calculated using the Exact Wilcoxon rank sum test (“wilcox.exact” function of the “exactRankTests” library in the R program with the options: “alternative=‘two.sided’, exact=TRUE, paired=F”). Each pair of the three groups was significantly differentiated based on its respective sequence distance (Types 1–2: *P*=0.0006536, Types 1–3: *P*=0.0001367, Types 2–3: *P*=0.003213, Fig. 1).

Since Japan is known to be rich in aquatic environments, with a large number of ponds, rivers, and spring waters, and has a large amount of rainfall throughout the year, we attempted to isolate new members of the family Mimiviridae from Japanese aquatic environments (Fig. 2), in addition to *M. shirakomae* and *M. kasaii*, and classify them in the family Mimiviridae using the SNS analysis described above. Water samples collected from the sea, a pond, and a river (Fig. 2) were subjected to the isolation of mimiviruses, as described previously (25). In the case of shellfish samples purchased from a market (an oyster and Asari clam [Japanese littleneck, Manila clam, *Ruditapes philippinarum*]), the body of the shellfish was crushed and mixed with 50 mL of PBS, and the supernatant liquid obtained after two centrifugal separations was collected (1st c.f.: 775×*g*, 10 min, 2nd c.f.: 1,158×*g*, 15 min). The following procedures that these samples were subjected to were the same as those for the water samples incubated in rice media, as described previously (25). The names, sampling places, exact sampling locations (longitude/latitude), and sample sources for the isolated viruses are shown in Fig. 2. The presence of nucleotide substitutions was examined for ten mimiviruses across four different regions by PCR and capillary sequencing. The primers used in this analysis are described in Table S3. Viral genomic DNA was purified from PYG culture media containing the viral particles by NucleoSpin Tissue XS (Macherey-Nagel GmbH and Co. KG, Düren, Germany). PCR assays were performed with the following steps: 94°C for 1 min, (98°C for 10 s, 55°C for 15 s, 68°C for 30 s)×35 cycles, 68°C for 7 min, and 4°C ∞, and the reaction was performed using Tks Gfelx DNA polymerase (TaKaRa Bio, Kusatsu, Japan). Sequence analyses were performed using TaKaRa Bio (Sanger sequencing reactions: BigDye Terminator v3.1 Cycle Sequencing Kit, Platform: Applied Biosystems 3730xl DNA Analyzer). This PCR approach is known as multi-locus sequencing typing (MLST) (18). We selected four regions for sequence analyses: regions A, B, C, and D; region A/C may classify type 1 and 2/3 viruses, and region B/D may classify type 2 and 1/3 viruses (Fig. 1 and Table S3). Regions B/D contain one SNS and regions A/C contain two juxtaposed SNSs; thus, six SNSs for each mimivirus were confirmed in this analysis (Table S3). The sequences of 4 out of 19 loci in the genomes of 9 new mimiviruses isolated from Japan were found to be identical (Data S4), and were classified into lineage A, genotype group type 2, similar to *M. shirakomae* and *M. kasaii*. This result suggests that mimiviruses isolated from Japan are closely related to each other or are putatively identical, with all belonging to lineage A, genotype group type 2. Thus, the local diversity of isolatable mimiviruses within a single community is not as high as that of viruses from the family Marseilleviridae; however, this situation may be overruled following further virus isolations. To confirm differences in local diversity, it will be necessary to compare these two families using fixed samples and environments in future analyses.

We previously isolated 16 members from the family Marseilleviridae from 3 sampling locations in Japan (4, 24). Among them, 13 members have distinct MCP genes with SNSs, and are classified into 4 groups: tokyovirus (1 isolate), kyotoviruses (5 isolates), hokutoviruses (2 isolates), and kashiwazakiviruses (5 isolates) (4, 24); the former two belonged to lineage A of the family Marseilleviridae, whereas the latter two groups belonged to lineage B (4). These findings suggest that the family Marseilleviridae has high local diversity for unknown reasons because several strains with different MCP gene sequences were isolated from a single sampling location (4). Furthermore, identical substitutions were found in the case of 11 mimivirus isolates from Japan (including 9 new mimiviruses, *M. shirakomae*, and *M. kasaii*) on 4 SNS loci, suggesting that members from the isolatable family Mimiviridae have lower divergence than members from the isolatable family Marseilleviridae.

In contrast to the pattern observed for the mimivirus genotypes (from lineage A) isolated from Japan, those from France appeared to be more diverse. *M. fauteuil*, *M. longchamps*, and *M. pointe-rouge 1*, which were isolated in Marseille, France, were classified into lineage A genotype group type 2, similar to the 11 Japanese mimiviruses, whereas *M. pointe-rouge 2*, *M. terra 2*, and *A. polyphaga lentillevirus* isolates were classified into lineage A genotype group type 3 (Fig. 1 and Table S2). Additionally, mimiviruses isolated from Brazil were classified into two groups: genotype group types 2 and 3. A metagenomic analysis of giant viruses in forest soils revealed that various giant viruses inhabit the soil environment (13). *Acanthamoeba*, which may be a natural host for giant viruses in nature, is known to be a common soil amoeba; when present in the soil environment, it has been shown to change the bacterial community in the rhizosphere of *Arabidopsis thaliana* (22), thereby indicating that it is alive in soil. Furthermore, *Acanthamoeba* spp. harboring mimivirus-like particles have been found in sewage sludge (23). Therefore, these classifications of mimiviruses may be loosely dependent on their sampling locations, and these differences may depend on the host distribution. For example, French type 2 mimiviruses were isolated from various environments including hospital water (*M. fauteuil*), decorative fountain water (*M*. *longchanps*), and sea water (*M. pointerouge* 1), whereas those of type 3 were isolated from sea water (*M. pointerouge* 2), soil (*M. terra 2*), and lens liquid (*A. polyphaga lentillevirus isolates lvs*) (Table S2). In contrast, Brazilian mimiviruses isolated from the Negro River were classified into genotype group type 2, while the Oyster virus and Kroon virus were classified into genotype group type 3 (Fig. 1 and Table S2), suggesting that isolatable mimivirus genotype groups inhabiting the same environment are likely to be identical or very similar to each other. Based on the multi-locus genotyping analysis focusing on SNS loci, we defined the genotype groups for the nine new mimiviruses isolated from various aquatic environments in Japan, such as freshwater lakes, rivers, beaches, and shellfishes (Fig. 2). The results showing that all mimiviruses isolated from several locations in Japan in the present study belong to lineage A and that they are all identical with regards to these variable SNS positions found in members from lineage A of the family Mimiviridae were unexpected. Further studies are needed to elucidate the mechanisms underlying the expansion of Mimiviridae lineage A type 2 on Japanese islands.

## SUPPLEMENTARY MATERIAL



## Figures and Tables

**Fig. 1:**
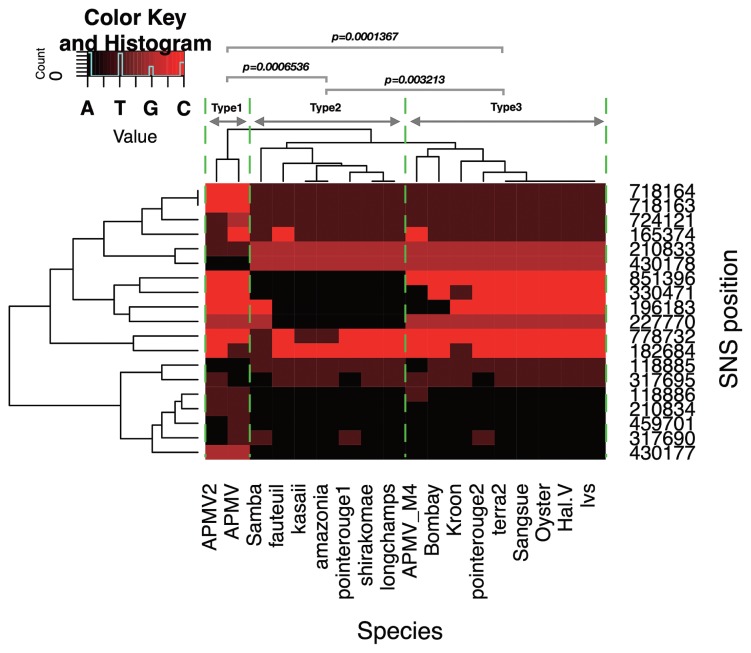
Heat map cluster analysis of SNS species harbored in mimiviruses from lineage A in Japan. Nineteen loci of SNSs harbored by mimiviruses isolated from Japan are clustered among lineage A. “SNS position” on the right side of the graph indicates physical positions on the APMV genome (NC_014649). Cell colors of the heat map correspond to the nucleotide species shown on the top left side of the graph. The green arrows show the subgroup of lineage A based on the clusters in rows, which are separated by two yellow dotted lines (types 1, 2, and 3). Virus names are abbreviated at the bottom of the graph (“species”). *P*-value: Pairwise distance of each genotype against Type 1, grouped with Type 1–3 genotype groups (Exact Wilcoxon rank sum test).

**Fig. 2:**
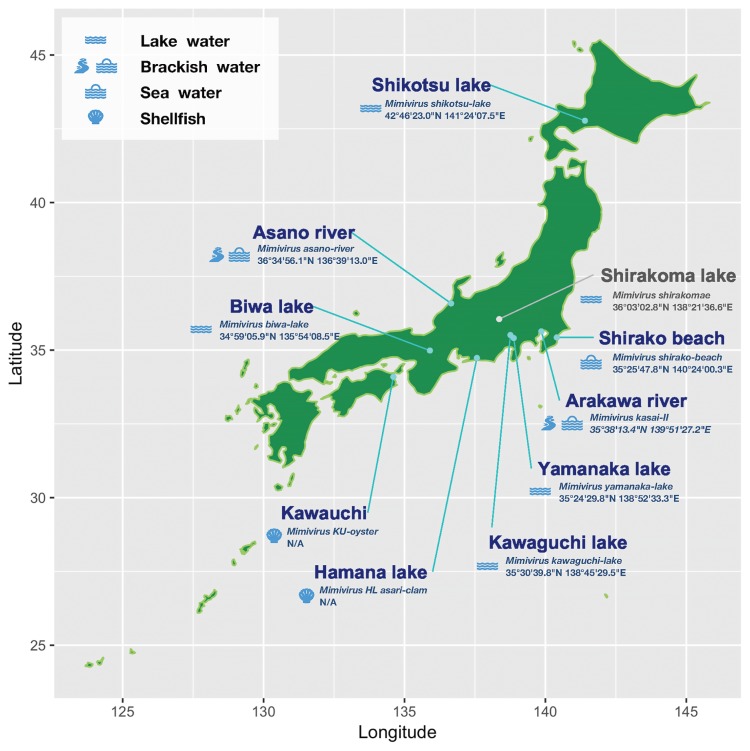
A sampling location map for lineage A, genotype group type 2 mimiviruses in Japan. The latitude/longitude of nine different locations on the Japanese Islands (light-blue) with corresponding virus names are shown, in addition to Shirakoma lake (grey), from which mimiviruses were isolated. Sample types are displayed as icons matched to the box on the top left. Please refer to the text for more details. Mimiviruses from biological samples for which the exact sampling locations are not known (KU-oyster and HL-asari-clam) are labeled as “N/A.” *Mimivirus shirakomae*, which we previously reported (25), is listed as a reference.
