# Supplementary material for: Distribution of SNSs in Mimivirus Genomes and the Classification of Mimiviruses Isolated from Japan
Source: Microbes Environ. 2019 Dec 27;34(4):451–5. doi: 10.1264/jsme2.ME19077 (PMC6934397; doi:10.1264/jsme2.ME19077)
Supplement: Supplementary file 1 [file 34_451_s1.pdf]

Supplementary Materials for

Distribution of SNSs in Mimivirus Genomes and the Classification of Mimiviruses Isolated from Japan

Motohiro Akashi[1]*, and Masaharu Takemura[1]

[1]*Laboratory of Biology, Department of Liberal Arts, Faculty of Science, Tokyo University of Science, Kagurazaka 1-3, Shinjuku, Tokyo 162-8601, Japan*

*Corresponding author. E-mail: motoa@rs.tus.ac.jp;

Tel: +81-3-5228-8373; FAX: +81-3-5228-8373.

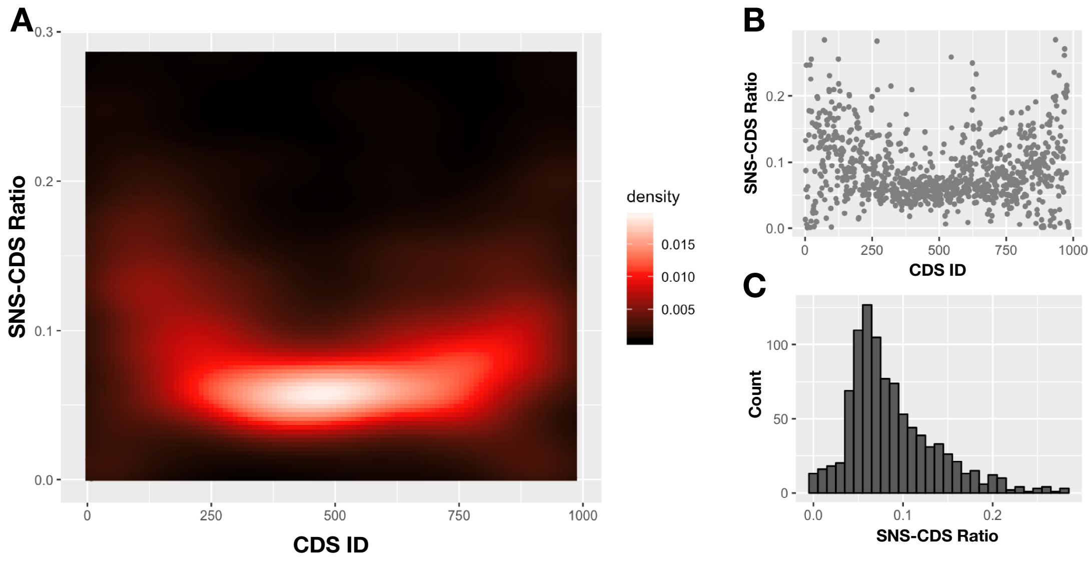

Fig. S1. Correlation of SNS-CDS ratios based on CDS position on the APMV genome. SNS-CDS ratios were calculated for each of the CDSs of APMV using the following formula: [SNS-CDS ratio = no. of SNS sp./CDS length (bp)]. "CDS ID" indicates the serial number of every CDS from the 5' to the 3' end of the APMV genome. (**A and B)** SNS-CDS ratio plotted against CDS ID. "density": density of the dot plot showing on the graph A. (**C**) Histogram of SNS-CDS Ratio.

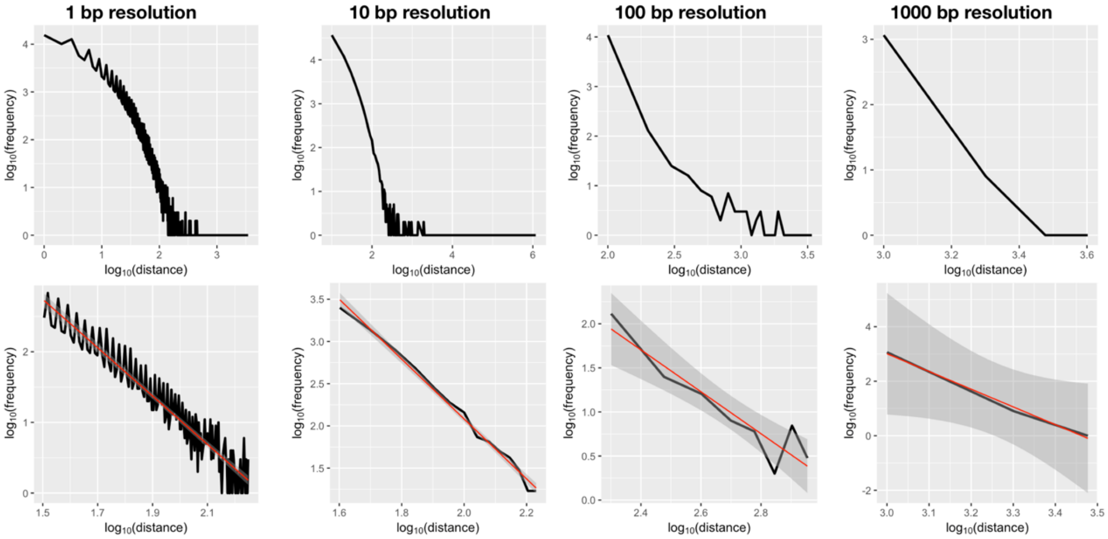

Resolution	Formula	R^2	Interval
1 bp	y=7.9-3.4x	0.896	1.5 =< $\log_{10}$(distance) < 2.25
10 bp	y=9.2-3.5x	0.993	1.5 =< $\log_{10}$(distance) < 2.25
100 bp	y=7.4-2.4x	0.871	2.25 =< $\log_{10}$(distance) < 3.0
1000 bp	y=23-6.5x	0.993	3.0 =< $\log_{10}$(distance) < 3.5

Fig. S2. SNS distribution patterns on the APMV genome. The distribution of SNSs shown under four resolutions; 1 bp, 10 bp, 100 bp and 1 kb. The first row (top) illustrate the whole region, and second row (bottom) illustrates the enlarged area of interest. The red straight lines are the approximate lines. The table below indicates the formulae of the approximate lines and their coefficient of determination (R^2). "Interval" in the table indicates the $\log_{10}$(distance) intervals of the enlarged graphs.

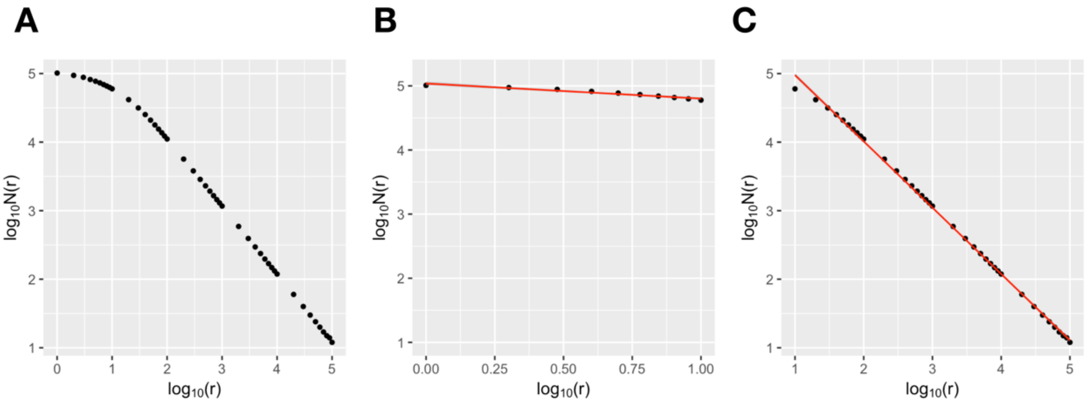

Formula	R^2	Interval
y=5-0.23x	0.948	0 =< $\log_{10}$(r) <= 1
y=5.9-0.97x	0.999	1 =< $\log_{10}$(r) <= 5

Fig. S3. Fractal nature of SNS distribution pattern on the APMV genome. (**A**) The distribution of SNSs indicates two fractal regions in low resolution and high resolution. (**B and C**) Both regions are enlarged, at resolution ≤10 bp (B) and ≥10 bp (C). If the resolution is more than 10 bp, then the shape of the regions including SNSs is fractal. The fractal dimension is 0.97. If the resolution is less than 10 bp, regions that include SNSs also have a fractal-structure. The fractal dimension is 0.23. The red straight lines in the figures are the approximate lines. The table below indicates the formula of the approximate lines and its coefficient of determination (R^2). "Interval" in the table indicate the $\log_{10}$(r) intervals of the enlarged pictures displayed.

Table S1. SNS analysis summary.

Type					Count (%)
**Substitution**					207,822（82.34%）
	**A-C**	6,202（2.46%）	**G-A**	38,285（15.17%）	
	**A-G**	34,710（13.75%）	**G-C**	2,577（1.02%）	
	**A-T**	15,538（6.16%）	**G-T**	6,490（2.57%）	
	**T-A**	16,139（6.39%）	**C-A**	6,428（2.55%）	
	**T-C**	34,478（13.66%）	**C-G**	2,531（1.00%）	
	**T-G**	6,263（2.48%）	**C-T**	38,181（15.13%）	
**Insertion**					22,413（8.88%）
**Deletion**					22,147（8.78%）
				**Total**	252,382（100%）
No. of analysed *Mimiviridae*					37
Query sequence					*APMV*
No. of SNSs positions detected on query genome					101,786
Query's CDSs harboring SNSs / Total CDSs (%)					950 / 985 (96.9%)

Thirty-seven sets of *Mimiviridae* genomes were obtained from the NCBI database, and 36 species of *Mimiviridae* SNSs against APMV genome (NC_014649) were called.

Table S2. Sample sources and sampling locations of the lineage A Mimivirus.

Virus Species	Source	Country
Mimivirus pointerouge2	Sea water	Marseille (France)
Mimivirus terra2	Soil	Marseille (France)
Acanthamoeba polyphaga mimivirus strain Oyster	Oyster farmed in the Atlantic coast	Florianopolis (Brazil)
Acanthamoeba polyphaga mimivirus strain Kroon	Urban lake water	Minas Gerais (Brazil)
Acanthamoeba castellanii mamavirus strain Hal-V	Cooling tower water	Paris (France)
Acanthamoeba polyphaga lentillevirus isolate lvs	Lens liquid	Marseille (France)
Hirudovirus strain Sangsue	*Hirudo medicinalis*	Oued Sarrath (Tunisia)
Mimivirus Bombay isolate 1	Sewage water	Mumbai (India)
Mimivirus fauteuil	Hospital water	Marseille (France)
Acanthamoeba castellanii mimivirus strain: Mimivirus kasaii	Brackish water	Tokyo (Japan)
Acanthamoeba castellanii mimivirus strain: Mimivirus shirakomae	Lake water	Nagano (Japan)
Mimivirus longchamps	Decorative fountain water	Marseille (France)
Mimivirus amazonia	Negro River water	Amazon (Brazil)
Mimivirus pointerouge1	Sea water	Marseille (France)
Samba virus	Negro River water	Amazon (Brazil)
Acanthamoeba polyphaga mimivirus isolate M4	Cooling tower water	Bradford (UK)
Acanthamoeba polyphaga mimivirus 2	Cooling tower water	Bradford (UK)
Acanthamoeba polyphaga mimivirus	Cooling tower water	Bradford (UK)

Table S3. PCR primers for sub-typing the *Mimivirus* lineage A.

Region	Pos. 1	Pos. 2	Locus	Forward primer	Revers primer
A	210833	210834	211233 - 210433	5'- gaatgtttcgttgtcaatacca -3'	5'- acacgtcgcaaaacttgtca -3'
B	330471		330071 - 330871	5'- tgatccaaatattgttggtgttt -3'	5'- tggatcacttccaggaggttt -3'
C	718163	718164	717764 - 718564	5'- ttgacgacaaaatgagtgagc -3'	5'- cgtttgaaaacactaaatctccatc -3'
D	851396		850996 - 851796	5'- ggattgctaatggacaaactga -3'	5'- cattaagaactccacgactagtaaatc -3'

Four different regions, named “A”, “B”, “C”, and “D”, on the APMV genome (NC_014649) were analysed using these primers. Regions A and C harbour two adjoining SNSs. The numbers in the “Pos.1”, “Pos.2”, and “Locus” columns indicate the physical positions on the APMV genome. The intervals in the rows of “Locus” are the regions amplified using the specific primer sets.

Data S1: Analyzed virus genome accession ID list.

taxid	Virus species	Accession ID
554168	Acanthamoeba_castellanii_mamavirus_strain_Hal-V	JF801956.1
1899318	Acanthamoeba_castellanii_mimivirus_strain_Mimivirus_kasaii	AP017644.1
1899318	Acanthamoeba_castellanii_mimivirus_strain_Mimivirus_shirakomae	AP017645.1
1077221	Acanthamoeba_polyphaga_lentillevirus_isolate_lvs	AFYC01000001.1
1077221	Acanthamoeba_polyphaga_lentillevirus_isolate_lvs	AFYC01000002.1
1077221	Acanthamoeba_polyphaga_lentillevirus_isolate_lvs	AFYC01000003.1
1077221	Acanthamoeba_polyphaga_lentillevirus_isolate_lvs	AFYC01000004.1
1077221	Acanthamoeba_polyphaga_lentillevirus_isolate_lvs	AFYC01000005.1
1077221	Acanthamoeba_polyphaga_lentillevirus_isolate_lvs	AFYC01000006.1
1077221	Acanthamoeba_polyphaga_lentillevirus_isolate_lvs	AFYC01000007.1
1077221	Acanthamoeba_polyphaga_lentillevirus_isolate_lvs	AFYC01000008.1
1077221	Acanthamoeba_polyphaga_lentillevirus_isolate_lvs	AFYC01000009.1
1077221	Acanthamoeba_polyphaga_lentillevirus_isolate_lvs	AFYC01000010.1
1077221	Acanthamoeba_polyphaga_mimivirus	NC_014649.1
212035	Acanthamoeba_polyphaga_mimivirus_2	AY653733.1
212035	Acanthamoeba_polyphaga_mimivirus_isolate_M4	JN036606.1
212035	Acanthamoeba_polyphaga_mimivirus_strain_Kroon	KM982402.1
212035	Acanthamoeba_polyphaga_mimivirus_strain_Oyster	KM982401.1
1269028	Acanthamoeba_polyphaga_moumouvirus	NC_020104.1
2071566	Bandra_megavirus_isolate_KK-1	MG779292.1
2071566	Bandra_megavirus_isolate_KK-1	MG779293.1
2071566	Bandra_megavirus_isolate_KK-1	MG779294.1
2071566	Bandra_megavirus_isolate_KK-1	MG779295.1
2071566	Bandra_megavirus_isolate_KK-1	MG779296.1
2071566	Bandra_megavirus_isolate_KK-1	MG779297.1
2071566	Bandra_megavirus_isolate_KK-1	MG779298.1
2071566	Bandra_megavirus_isolate_KK-1	MG779299.1
2071566	Bandra_megavirus_isolate_KK-1	MG779300.1
2071566	Bandra_megavirus_isolate_KK-1	MG779301.1
2071566	Bandra_megavirus_isolate_KK-1	MG779302.1
2071566	Bandra_megavirus_isolate_KK-1	MG779303.1
2071566	Bandra_megavirus_isolate_KK-1	MG779304.1
2071566	Bandra_megavirus_isolate_KK-1	MG779305.1
2071566	Bandra_megavirus_isolate_KK-1	MG779306.1
2071566	Bandra_megavirus_isolate_KK-1	MG779307.1
2071566	Bandra_megavirus_isolate_KK-1	MG779308.1
2071566	Bandra_megavirus_isolate_KK-1	MG779309.1
2071566	Bandra_megavirus_isolate_KK-1	MG779310.1
2071566	Bandra_megavirus_isolate_KK-1	MG779311.1
2071566	Bandra_megavirus_isolate_KK-1	MG779312.1
2071566	Bandra_megavirus_isolate_KK-1	MG779313.1
2071566	Bandra_megavirus_isolate_KK-1	MG779314.1
2071566	Bandra_megavirus_isolate_KK-1	MG779315.1
2071566	Bandra_megavirus_isolate_KK-1	MG779316.1
2071566	Bandra_megavirus_isolate_KK-1	MG779317.1
2071566	Bandra_megavirus_isolate_KK-1	MG779318.1
2071566	Bandra_megavirus_isolate_KK-1	MG779319.1
2071566	Bandra_megavirus_isolate_KK-1	MG779320.1
2071566	Bandra_megavirus_isolate_KK-1	MG779321.1
2071566	Bandra_megavirus_isolate_KK-1	MG779322.1
2071566	Bandra_megavirus_isolate_KK-1	MG779323.1
2071566	Bandra_megavirus_isolate_KK-1	MG779324.1
2071566	Bandra_megavirus_isolate_KK-1	MG779325.1
2071566	Bandra_megavirus_isolate_KK-1	MG779326.1
2071566	Bandra_megavirus_isolate_KK-1	MG779327.1
2071566	Bandra_megavirus_isolate_KK-1	MG779328.1
2071566	Bandra_megavirus_isolate_KK-1	MG779329.1
2071566	Bandra_megavirus_isolate_KK-1	MG779330.1
2071566	Bandra_megavirus_isolate_KK-1	MG779331.1
2071566	Bandra_megavirus_isolate_KK-1	MG779332.1
2071566	Bandra_megavirus_isolate_KK-1	MG779333.1
2071566	Bandra_megavirus_isolate_KK-1	MG779334.1
2071566	Bandra_megavirus_isolate_KK-1	MG779335.1
2071566	Bandra_megavirus_isolate_KK-1	MG779336.1
2071566	Bandra_megavirus_isolate_KK-1	MG779337.1
2071566	Bandra_megavirus_isolate_KK-1	MG779338.1
2071566	Bandra_megavirus_isolate_KK-1	MG779339.1
2071566	Bandra_megavirus_isolate_KK-1	MG779340.1
2071566	Bandra_megavirus_isolate_KK-1	MG779341.1
2071566	Bandra_megavirus_isolate_KK-1	MG779342.1
2071566	Bandra_megavirus_isolate_KK-1	MG779343.1
2071566	Bandra_megavirus_isolate_KK-1	MG779344.1
2071566	Bandra_megavirus_isolate_KK-1	MG779345.1
2071566	Bandra_megavirus_isolate_KK-1	MG779346.1
2071566	Bandra_megavirus_isolate_KK-1	MG779347.1
2071566	Bandra_megavirus_isolate_KK-1	MG779348.1
2071566	Bandra_megavirus_isolate_KK-1	MG779349.1
2071566	Bandra_megavirus_isolate_KK-1	MG779350.1
2071566	Bandra_megavirus_isolate_KK-1	MG779351.1
2071566	Bandra_megavirus_isolate_KK-1	MG779352.1
2071566	Bandra_megavirus_isolate_KK-1	MG779353.1
2071566	Bandra_megavirus_isolate_KK-1	MG779354.1
2071566	Bandra_megavirus_isolate_KK-1	MG779355.1
2071566	Bandra_megavirus_isolate_KK-1	MG779356.1
2071566	Bandra_megavirus_isolate_KK-1	MG779357.1
2071566	Bandra_megavirus_isolate_KK-1	MG779358.1
2071566	Bandra_megavirus_isolate_KK-1	MG779359.1
2071566	Bandra_megavirus_isolate_KK-1	MG779360.1
2071566	Bandra_megavirus_isolate_KK-1	MG779361.1
2071566	Bandra_megavirus_isolate_KK-1	MG779362.1
2071566	Bandra_megavirus_isolate_KK-1	MG779363.1
2071566	Bandra_megavirus_isolate_KK-1	MG779364.1
2071566	Bandra_megavirus_isolate_KK-1	MG779365.1
2071566	Bandra_megavirus_isolate_KK-1	MG779366.1
2071566	Bandra_megavirus_isolate_KK-1	MG779367.1
2071566	Bandra_megavirus_isolate_KK-1	MG779368.1
2071566	Bandra_megavirus_isolate_KK-1	MG779369.1
2071566	Bandra_megavirus_isolate_KK-1	MG779370.1
2071566	Bandra_megavirus_isolate_KK-1	MG779371.1
2071566	Bandra_megavirus_isolate_KK-1	MG779372.1
2071566	Bandra_megavirus_isolate_KK-1	MG779373.1
2071566	Bandra_megavirus_isolate_KK-1	MG779374.1
2071566	Bandra_megavirus_isolate_KK-1	MG779375.1
2071566	Bandra_megavirus_isolate_KK-1	MG779376.1
2071566	Bandra_megavirus_isolate_KK-1	MG779377.1
2071566	Bandra_megavirus_isolate_KK-1	MG779378.1
2071566	Bandra_megavirus_isolate_KK-1	MG779379.1
2071566	Bandra_megavirus_isolate_KK-1	MG779380.1
2071566	Bandra_megavirus_isolate_KK-1	MG779381.1
2071566	Bandra_megavirus_isolate_KK-1	MG779382.1
2071566	Bandra_megavirus_isolate_KK-1	MG779383.1
2071566	Bandra_megavirus_isolate_KK-1	MG779384.1
2071566	Bandra_megavirus_isolate_KK-1	MG779385.1
2071566	Bandra_megavirus_isolate_KK-1	MG779386.1
2071566	Bandra_megavirus_isolate_KK-1	MG779387.1
2071566	Bandra_megavirus_isolate_KK-1	MG779388.1
2071566	Bandra_megavirus_isolate_KK-1	MG779389.1
2071566	Bandra_megavirus_isolate_KK-1	MG779390.1
2071566	Bandra_megavirus_isolate_KK-1	MG779391.1
2071566	Bandra_megavirus_isolate_KK-1	MG779392.1
2071566	Bandra_megavirus_isolate_KK-1	MG779393.1
2071566	Bandra_megavirus_isolate_KK-1	MG779394.1
2071566	Bandra_megavirus_isolate_KK-1	MG779395.1
2071566	Bandra_megavirus_isolate_KK-1	MG779396.1
2071566	Bandra_megavirus_isolate_KK-1	MG779397.1
2071566	Bandra_megavirus_isolate_KK-1	MG779398.1
2071566	Bandra_megavirus_isolate_KK-1	MG779399.1
2071566	Bandra_megavirus_isolate_KK-1	MG779400.1
2071566	Bandra_megavirus_isolate_KK-1	MG779401.1
2071566	Bandra_megavirus_isolate_KK-1	MG779402.1
2071566	Bandra_megavirus_isolate_KK-1	MG779403.1
2071566	Bandra_megavirus_isolate_KK-1	MG779404.1
2071566	Bandra_megavirus_isolate_KK-1	MG779405.1
2071566	Bandra_megavirus_isolate_KK-1	MG779406.1
2071566	Bandra_megavirus_isolate_KK-1	MG779407.1
2071566	Bandra_megavirus_isolate_KK-1	MG779408.1
2071566	Bandra_megavirus_isolate_KK-1	MG779409.1
2071566	Bandra_megavirus_isolate_KK-1	MG779410.1
1420594	Hirudovirus_strain_Sangsue	KF493731.1
1128143	Megavirus terra1	KF527229.1
1420594	Megavirus_avenue9	LN867403.1
1420594	Megavirus_battle43	LN868256.1
1420594	Megavirus_bus	LN868539.1
1094892	Megavirus_chiliensis	NC_016072.1
1128140	Megavirus_courdo11	JX975216.1
1128141	Megavirus_courdo5	LN868540.1
1235314	Megavirus_lba_isolate_LBA111	JX885207.1
1643508	Megavirus_montpellier	LN868518.1
1643296	Megavirus_shan	LN868519.1
1643296	Megavirus_shan	LN868520.1
1643296	Megavirus_T1	LN868521.1
1643296	Megavirus_T1	LN868522.1
1643296	Megavirus_T1	LN868523.1
1643296	Megavirus_T1	LN868524.1
1643296	Megavirus_T1	LN868525.1
1643296	Megavirus_T1	LN868526.1
1643296	Megavirus_T4	LN869536.1
1643296	Megavirus_T4	LN869537.1
1128151	Mimivirus terra2	NC_023639.1
1643296	Mimivirus_amazonia	LN867402.1
1835008	Mimivirus_Bombay_isolate_1	KU761889.1
1128144	Mimivirus_fauteuil	LN871163.1
1128147	Mimivirus_longchamps	LN871173.1
1128149	Mimivirus_pointerouge1	LN871174.1
1128150	Mimivirus_pointerouge2	LN871172.1
1643295	Moumouvirus_battle49	LN871164.1
1643295	Moumouvirus_battle49	LN871165.1
1643295	Moumouvirus_battle49	LN871166.1
1643295	Moumouvirus_battle49	LN871167.1
1643295	Moumouvirus_battle49	LN871168.1
1643295	Moumouvirus_battle49	LN871169.1
1643295	Moumouvirus_battle49	LN871170.1
1643295	Moumouvirus_battle49	LN871171.1
1247379	Moumouvirus_goulette	KC008572.1
1842663	Powai_lake_megavirus_isolate_1	KU877344.1
1461100	Samba_virus	KF959826.2
1461100	Tupanvirus_isolate_deep_ocean	MF405918.1
2094720	Tupanvirus_isolate_soda_lake	KY523104.1

Data S2: SNSs and Amino acid substitiutions of the CDSs in the Mimivirus of Japan.

query genome position	query nt	subject nt	Virus	locus_tag	query_codon	subject_codon	query_aa	subject_aa	triplet_pos	aa_locus	Conserved_domain	protein_id
210834	T	A	kasaii&shirakomae	MIMI_gp0194	taA	taT	Stop	Tyr	3	88	N/A	YP_003986668.1
227770	G	A	kasaii&shirakomae	MIMI_gp0208	Gat	Aat	Asp	Asn	1	85	NAD(P)/FAD-dependent_oxidoreductas	YP_003986682.1
330471	C	A	kasaii&shirakomae	MIMI_gp0287	Gat	Tat	Leu	Ile	1	546	N/A	YP_003986761.1
459701	T	A	kasaii&shirakomae	MIMI_gp0383	Tta	Ata	Leu	Tyr	1	66	YqaJ_domain-containing_protein	YP_003986857.1
718163	C	T	kasaii&shirakomae	MIMI_gp0575	ttC	ttT	Phe	Phe	3	5	N/A	YP_003987048.1
718164	C	T	kasaii&shirakomae	MIMI_gp0575	Cat	Tat	His	Tyr	1	6	N/A	YP_003987048.1
724121	G	T	kasaii&shirakomae	MIMI_gp0581	aCt	aAt	Thr	Asn	2	147	TPD	YP_003987054.1
778732	C	T	kasaii	MIMI_gp0628	Cca	Tca	Pro	Ser	1	17	N/A	YP_003987100.1
851396	C	A	kasaii&shirakomae	MIMI_gp0697	aCt	aAt	Thr	Leu	2	197	NHL	YP_003987168.1

Data S3-1: C-scores of Estimated Structual Homologoues of the CDSs harboring SNSs in Mimivirus of Japan.

Locus Tag	C-score	Estimated TM-score	Estimated RMSD
MIMI_gp0194	-3.35	0.34±0.12	11.1±4.6Å
MIMI_gp0208	-0.31	0.67±0.13	8.0±4.4Å
MIMI_gp0287	-1.1	0.58±0.14	10.2±4.6Å
MIMI_gp0383	-0.99	0.59±0.14	9.8±4.6Å
MIMI_gp0575	-2.94	0.38±0.13	14.6±3.6Å
MIMI_gp0581	-4.68	0.23±0.06	17.9±2.5Å
MIMI_gp0628	-3.92	0.29±0.09	13.1±4.2Å
MIMI_gp0697	-1.17	0.57±0.15	9.5±4.6Å

C-score : Confidence score for estimating the quality of predicted models from “-5” to “2”.

Data S3-2: Top 3 Protein Functions of Estimated Structual Homologoues of the CDSs harboring SNSs in Mimivirus of Japan.

Locus Tag	Name	PDBID	Organism	Note
MIMI_gp0194	TT1805	1WN9	Thermus thermophilus strain HB8	hypothetical protein (TT1805)
MIMI_gp0194	PARN	3D45	Mus musculus	Poly(A)-specific ribonuclease (PARN).
MIMI_gp0194	Tob-hCaf1	2D5R	Homo sapiens	RNase D.
MIMI_gp0208	L-amino acid oxidase	3KVE	Vipera ammodytes ammodytes	L-amino acid oxidase.
MIMI_gp0208	LAAO	2IID	Calloselasma rhodostoma	L-amino acid oxidase.
MIMI_gp0208	AHPLAAO	1REO	Gloydius halys	L-amino acid oxidase.
MIMI_gp0287	V-type proton ATPase catalytic subunit A	5VOX	Saccharomyces cerevisiae	V-type proton ATPase.
MIMI_gp0287	V-type proton ATPase subunit c	6C6L	Saccharomyces cerevisiae	Vacuolar ATPase VoProton Channel.
MIMI_gp0287	V-type proton ATPase subunit a	5TJ5	Saccharomyces cerevisiae	Membrane-embedded motor of a eukaryotic V-ATPase.
MIMI_gp0383	Protein unc-13 homolog A	4Y21	Rattus norvegicus	Munc13-1 MUN domain.
MIMI_gp0383	Protein unc-13 homolog A	5UE8	Rattus norvegicus	Munc13-1 C1C2BMUN domain.
MIMI_gp0383	Exocyst complex component SEC3	5YFP	Saccharomyces cerevisiae	Exocyst Complex.
MIMI_gp0575	NUCLEOPORIN NUP43	5A9Q	Homo sapiens	Human nuclear pore.
MIMI_gp0575	Protein transport protein SEC13	3IKO	Saccharomyces cerevisiae	Sec13 nucleoporin protein.
MIMI_gp0575	Nucleoporin NIC96	5HB2	Chaetomium thermophilum	Chaetomium thermophilum Nic96 SOL.
MIMI_gp0581	BETA-KETOACYL SYNTHASE (FabY)	4CW4	Pseudomonas aeruginosa	Noncanonical ketosynthase FabY
MIMI_gp0581	GLUCOSYLCERAMIDASE	5FJS	Thermoanaerobacterium xylanolyticum	Human glucocerebrosidase 2 (GBA2).
MIMI_gp0581	Fatty acid synthase subunit alpha	2PFF	Saccharomyces cerevisiae	Fatty acid synthase subunit alpha.
MIMI_gp0628	Acyl-CoA thioester hydrolase ybgC	3GRH	Escherichia coli	Outer membrane lipoprotein YbhC.
MIMI_gp0628	Pectinesterase 1	1XG2	Solanum lycopersicum	Pectin methylesterase.
MIMI_gp0628	Thylakoid lumenal 15 kDa protein 1, chloroplastic	3N90	Arabidopsis thaliana	AT2G44920, a pentapeptide repeat protein from Arabidopsis thaliana thylakoid lumen.
MIMI_gp0697	E3 ubiquitin-protein ligase TRIM71	6FPT	Danio rerio	Danio rerio Lin41 filamin-NHL domains
MIMI_gp0697	Peptidyl-glycine alpha-amidating monooxygenase	3FW0	Rattus norvegicus	Peptidyl-alpha-hydroxyglycine alpha-Amidating Lyase (PAL)
MIMI_gp0697	Uncharacterized protein	3HRP	Bacteroides thetaiotaomicron	unknown function (NP_812590.1)

Data S4. Sequences of four of 6 loci on genome of new 9 mimivirus isolated from Japan.

(A)CLUSTAL format alignment of APMV genome (210793-210853) by MAFFT L-INS-i (v7.222)

```
APMV               ttcaaataaccattttgaaaaatatatttgtagtacattcttaattatacaatgaacta
asano-river        ttcaaataaccattttgaaaaatatatttgtagtacattcgataattatacaatgaacta
biwa-lake          ttcaaataaccattttgaaaaatatatttgtagtacattcgataattatacaatgaacta
HL asari-clam      ttcaaataaccattttgaaaaatatatttgtagtacattcgataattatacaatgaacta
kasai-II           ttcaaataaccattttgaaaaatatatttgtagtacattcgataattatacaatgaacta
kawaguchi-lake     ttcaaataaccattttgaaaaatatatttgtagtacattcgataattatacaatgaacta
KU-oyster          ttcaaataaccattttgaaaaatatatttgtagtacattcgataattatacaatgaacta
shikotsu-lake      ttcaaataaccattttgaaaaatatatttgtagtacattcgataattatacaatgaacta
shirako-beach      ttcaaataaccattttgaaaaatatatttgtagtacattcgataattatacaatgaacta
shirakomae         ttcaaataaccattttgaaaaatatatttgtagtacattcgataattatacaatgaacta
yamanaka-lake      ttcaaataaccattttgaaaaatatatttgtagtacattcgataattatacaatgaacta
                   ****************************************  ******************
```

(B) CLUSTAL format alignment of APMV genome (330431-330491) by MAFFT L-INS-i (v7.222)

```
APMV               tggtctatgtacttctgtttttattctaatgggcttcttatcgggatatttttgtagatt
asano-river        tggtctatgtacttctgtttttattctaatgggcttcttatagggatatttttgtagatt
biwa-lake          tggtctatgtacttctgtttttattctaatgggcttcttatagggatatttttgtagatt
HL asari-clam      tggtctatgtacttctgtttttattctaatgggcttcttatagggatatttttgtagatt
kasai-II           tggtctatgtacttctgtttttattctaatgggcttcttatagggatatttttgtagatt
kawaguchi-lake     tggtctatgtacttctgtttttattctaatgggcttcttatagggatatttttgtagatt
KU-oyster          tggtctatgtacttctgtttttattctaatgggcttcttatagggatatttttgtagatt
shikotsu-lake      tggtctatgtacttctgtttttattctaatgggcttcttatagggatatttttgtagatt
shirako-beach      tggtctatgtacttctgtttttattctaatgggcttcttatagggatatttttgtagatt
shirakomae         tggtctatgtacttctgtttttattctaatgggcttcttatagggatatttttgtagatt
yamanaka-lake      tggtctatgtacttctgtttttattctaatgggcttcttatagggatatttttgtagatt
                   ***************************************** ******************
```

(C) CLUSTAL format alignment of APMV genome (718124-718184) by MAFFT L-INS-i (v7.222)

```
APMV               acaatctactatttattaaaataatatggaaaccgtattccatcaaaagaaaaatgccac
asano-river        acaatctactatttattaaaataatatggaaaccgtattttatcaaaagaaaaatgccac
biwa-lake          acaatctactatttattaaaataatatggaaaccgtattttatcaaaagaaaaatgccac
HL asari-clam      acaatctactatttattaaaataatatggaaaccgtattttatcaaaagaaaaatgccac
kasai-II           acaatctactatttattaaaataatatggaaaccgtattttatcaaaagaaaaatgccac
kawaguchi-lake     acaatctactatttattaaaataatatggaaaccgtattttatcaaaagaaaaatgccac
KU-oyster          acaatctactatttattaaaataatatggaaaccgtattttatcaaaagaaaaatgccac
shikotsu-lake      acaatctactatttattaaaataatatggaaaccgtattttatcaaaagaaaaatgccac
shirako-beach      acaatctactatttattaaaataatatggaaaccgtattttatcaaaagaaaaatgccac
yamanaka-lake      acaatctactatttattaaaataatatggaaaccgtattttatcaaaagaaaaatgccac
                   ***************************************..*******************
```

(D) CLUSTAL format alignment of APMV genome (851356-851416) by MAFFT L-INS-i (v7.222)

```
APMV               gatcctcttgttagtttccttgtacttaatgaacaattgactggtgaaattcatgtattt
asano-river        gatcctcttgttagtttccttgtacttaatgaacaattgaatggtgaaattcatgtattt
biwa-lake          gatcctcttgttagtttccttgtacttaatgaacaattgaatggtgaaattcatgtattt
HL asari-clam      gatcctcttgttagtttccttgtacttaatgaacaattgaatggtgaaattcatgtattt
kasai-II           gatcctcttgttagtttccttgtacttaatgaacaattgaatggtgaaattcatgtattt
kawaguchi-lake     gatcctcttgttagtttccttgtacttaatgaacaattgaatggtgaaattcatgtattt
KU-oyster          gatcctcttgttagtttccttgtacttaatgaacaattgaatggtgaaattcatgtattt
shikotsu-lake      gatcctcttgttagtttccttgtacttaatgaacaattgaatggtgaaattcatgtattt
shirako-beach      gatcctcttgttagtttccttgtacttaatgaacaattgaatggtgaaattcatgtattt
yamanaka-lake      gatcctcttgttagtttccttgtacttaatgaacaattgaatggtgaaattcatgtattt
                   **************************************** *******************
```
